# Supplementary material for: Adolescent boys’ sociocultural beliefs and attitudes toward menstruation in selected high schools in Ghana: Mediation and moderation effect of knowledge
Source: PLOS Glob Public Health. 2025 Jun 5;5(6):e0004354. doi: 10.1371/journal.pgph.0004354 (PMC12140258; doi:10.1371/journal.pgph.0004354)
Supplement: S1 Table — This table presents responses to knowledge-based questions assessing the understanding of menstruation among adolescent boys (N = 431). (DOCX) [file pgph.0004354.s001.docx]

**S1 Table: Adolescent boys Knowledge on Menstruation and Menstrual-related issues**

| **Statement** | **Frequency** | **Percentage** |
| --- | --- | --- |
| **﻿What is menstruation?** |  |  |
| The monthly shedding of the uterine lining | 209 | 48.5 |
| The process of releasing an egg from the ovary | 187 | 43.4 |
| The release of hormones that cause mood swings | 21 | 4.9 |
| I do not know | 14 | 3.3 |
| **﻿Which of the following describes what menstruation is?** |  |  |
| A curse | 2 | 0.5 |
| A normal biological process | 340 | 78.9 |
| A reason girls should stay home | 10 | 2.3 |
| Dirty and something girls should hide | 71 | 16.5 |
| I do not know | 8 | 1.9 |
| **﻿Within what age range do most girls get their first period?** |  |  |
| 10-17 years | 354 | 82.1 |
| Less than 10 years | 8 | 1.9 |
| More than 17 years | 56 | 13.0 |
| I do not know | 13 | 3.0 |
| **﻿How many days does a normal menstruation last?** |  |  |
| 2-7 days | 288 | 66.8 |
| 28 days | 29 | 6.7 |
| 30 days | 11 | 2.6 |
| 7-14 days | 71 | 16.5 |
| I do not know | 32 | 7.4 |
| **﻿Which part of the body does period blood flow out from?** |  |  |
| Bladder | 2 | 0.5 |
| Intestines | 1 | 0.2 |
| Uterus/womb and through the vagina | 74 | 17.2 |
| Vagina | 348 | 80.7 |
| I do not know | 6 | 1.4 |
| **﻿Which of the following show that a girl menstruating might need medical attention?** |  |  |
| Change in appetite | 29 | 6.7 |
| Heavy bleeding with clots | 295 | 68.5 |
| Mild cramps | 70 | 16.2 |
| Slight mood swings | 27 | 6.3 |
| I do not know | 10 | 2.3 |
| **﻿When a girl or woman begins having her menses, what does it mean?** |  |  |
| Can become pregnant if she has unprotected sex | 371 | 86.1 |
| Cannot get pregnant | 28 | 6.5 |
| Is Pregnant | 9 | 2.1 |
| None of the above | 17 | 3.9 |
| I do not know | 6 | 1.4 |
| **﻿What should a girl do if her period is painful/ irregular?** |  |  |
| It's normal and she just has to deal with it | 58 | 13.5 |
| She did something wrong | 5 | 1.2 |
| She should not do anything | 2 | 0.5 |
| She should see a doctor | 359 | 83.3 |
| She's lying about the pain | 3 | 0.7 |
| I do not know | 4 | 0.9 |
| **﻿Menstruation usually stops around what age range?** |  |  |
| 20-30 years | 23 | 5.3 |
| 30-40 years | 62 | 14.4 |
| 45-58 years | 232 | 53.8 |
| 60+ years | 97 | 22.5 |
| I do not know | 17 | 3.9 |
| **﻿Having a period happens for what reason?** |  |  |
| The body is preparing for pregnancy | 170 | 39.4 |
| The body releases toxins | 77 | 17.9 |
| The uterus is cleaning itself out | 132 | 30.6 |
| None of the above | 18 | 4.2 |
| I do not know | 34 | 7.9 |
| **﻿When girls have signs like cramps, bloats and mood swings before their period comes what does it mean?** |  |  |
| Normal parts of having a period | 191 | 44.3 |
| Not real and exaggerated | 2 | 0.5 |
| Not real and they are just making it sound too much | 9 | 2.1 |
| Should not be discussed | 5 | 1.2 |
| Signs something is wrong | 195 | 45.2 |
| I do not know | 29 | 6.7 |
| **﻿Which of the following best describes the use of menstruation products like pads, and tampons to absorb period blood?** |  |  |
| Not allowed | 2 | 0.5 |
| They are Optional | 87 | 20.2 |
| They are Recommended | 195 | 45.2 |
| They are Required | 138 | 32.0 |
| I do not know | 9 | 2.1 |
| **﻿Which of the following best describes the health benefits of getting a period?** |  |  |
| There are no benefits | 35 | 8.1 |
| Building the uterine lining | 86 | 20.0 |
| Regulation of hormones | 111 | 25.8 |
| Both B and C | 157 | 36.4 |
| I do not know | 42 | 9.7 |
