# Supplementary material for: Adolescent boys’ sociocultural beliefs and attitudes toward menstruation in selected high schools in Ghana: Mediation and moderation effect of knowledge
Source: PLOS Glob Public Health. 2025 Jun 5;5(6):e0004354. doi: 10.1371/journal.pgph.0004354 (PMC12140258; doi:10.1371/journal.pgph.0004354)
Supplement: S2 Table — This table displays how participants (N = 431) perceive and experience cultural and familial beliefs surrounding menstruation. (DOCX) [file pgph.0004354.s002.docx]

**S2 Table: Sociocultural beliefs about menstruation among adolescent boys in the Volta region**

|  | **N=431** | | | | |
| --- | --- | --- | --- | --- | --- |
| **Statement** | **Strongly Disagree** | **Disagree** | **Neutral** | **Agree** | **Strongly Agree** |
| ﻿In my family/culture, it is taboo to talk about periods | 120 (27.8) | 196 (45.5) | 28 (6.5) | 64 (14.9) | 23 (5.3) |
| ﻿I was taught that menstruation is something private that should be hidden | 75 (17.4) | 127 (29.5) | 26 (6.0) | 153(35.5) | 50(11.6) |
| ﻿In my family/culture, women are considered unclean/dirty during menstruation | 95 (22.0) | 138 (32.0) | 22 (5.1) | 134(31.1) | 42 (9.7) |
| ﻿A sister/female relative was made to feel ashamed/bad about getting her first period | 106 (24.6) | 165 (38.3) | 29 (6.7) | 105(24.4) | 26 (6.0) |
| ﻿My family/culture has a ceremony or tradition associated with a girl getting her first period | 90 (20.9) | 155(36.0) | 42 (9.7) | 108(25.1) | 36 (8.4) |
| ﻿In my family/culture, women do not talk about periods with men | 61 (14.2) | 159 (36.9) | 37 (8.6) | 128(29.7) | 46 (10.7) |
| ﻿I can talk openly about periods with my mother/other female relatives | 26 (6.0) | 64 (14.9) | 20 (4.6) | 204(47.3) | 117 (27.2) |
| ﻿My family/culture has certain norms about what women can/cannot do while menstruating | 43 (10.0) | 84 (19.5) | 25 (5.8) | 178(41.3) | 101 (23.4) |
| ﻿Women in my family/culture rely on each other for guidance about periods rather than getting information elsewhere | 18 (4.2) | 80 (18.6) | 25 (5.8) | 234(54.3) | 74 (17.2) |
| ﻿In my culture/family, women are isolated during menstruation for spiritual or religious reasons | 130 (30.2) | 165 (38.3) | 30 (7.0) | 69 (16.0) | 37 (8.6) |
| ﻿My family's/culture's views on menstruation made me feel ashamed growing up | 103 (23.9) | 217 (50.4) | 29 (6.7) | 59 (13.7) | 23 (5.3) |
| ﻿Menstruation is a taboo topic for both men and women in my culture/family | 141 (32.71) | 213 (49.4) | 23 (5.3) | 39 (9.1) | 15 (3.5) |
| ﻿I make an effort to educate others about stigmatized aspects of cultural beliefs around menstruation | 52 (12.1) | 108 (25.1) | 39 (9.1) | 155(36.0) | 77 (17.9) |
