# Supplementary material for: Adolescent boys’ sociocultural beliefs and attitudes toward menstruation in selected high schools in Ghana: Mediation and moderation effect of knowledge
Source: PLOS Glob Public Health. 2025 Jun 5;5(6):e0004354. doi: 10.1371/journal.pgph.0004354 (PMC12140258; doi:10.1371/journal.pgph.0004354)
Supplement: S3 Table — This table presents statements on the attitudes of adolescent boys (N = 431) concerning menstruation. (DOCX) [file pgph.0004354.s003.docx]

**S3 Table: Adolescent boys’ attitudes towards menstruation and menstrual-related issues**

|  | **N=431** | | | | |
| --- | --- | --- | --- | --- | --- |
| **Statement** | **Strongly Disagree**  **n (%)** | **Disagree**  **n (%)** | **Neutral**  **n (%)** | **Agree**  **n (%)** | **Strongly Agree**  **n (%)** |
| ﻿Menstruation is a natural and normal biological process | 5 (1.2) | 2 (0.5) | 3 (0.7) | 191 (44.3) | 230(53.4) |
| ﻿Menstruation is a taboo topic | 172 (39.9) | 204 (47.3) | 7 (1.6) | 37 (8.6) | 11 (2.5) |
| ﻿Menstruation is only a female issue/matter and does not concern males | 107 (24.8) | 179 (41.5) | 15 (3.5) | 82 (19.0) | 48 (11.1) |
| ﻿I would feel comfortable buying menstrual products like pads for a female friend or family member | 29 (6.7) | 40 (9.3) | 14 (3.3) | 223 (51.7) | 125(29.0) |
| ﻿Society should break the stigma and silence/ discrimination/bad perception around menstruation | 37 (8.6) | 69 (16.0) | 15 (3.5) | 180 (41.8) | 130(30.2) |
| ﻿There should be more education about menstruation targeted especially for boys and men | 21 (4.9) | 42 (9.7) | 12 (2.8) | 228 (52.9) | 128(29.7) |
| ﻿Menstruation is nasty/unpleasant/not nice | 71 (16.5) | 109 (25.3) | 42 (9.7) | 157 (36.4) | 52 (12.1) |
| ﻿Girls/women should try to hide the fact that they are on their periods | 72 (16.7) | 147 (34.1) | 24 (5.6) | 150 (34.8) | 38 (8.8) |
| ﻿Girls should be allowed to miss school if they have severe period pain or symptoms | 31 (7.2) | 76 (17.6) | 29 (6.7) | 208 (48.3) | 87 (20.2) |
| ﻿I would tell a girl who had a visible blood stain on her clothes from her period | 35 (8.1) | 13 (3.0) | 12 (2.8) | 245 (56.8) | 126(29.2) |
| ﻿Women/girls often talk too much about [exaggerate] the pain and discomfort they get from menstruation | 28 (6.5) | 119(27.6) | 39(9.1) | 190 (44.1) | 55 (12.8) |
| ﻿I would feel comfortable discussing menstruation with a girlfriend, family member, or female friend | 21 (4.9) | 71 (16.5) | 22 (5.1) | 219 (50.8) | 98 (22.7) |
| ﻿Menstruation is an excuse some women use not to do activities | 39 (9.1) | 96 (22.3) | 26 (6.0) | 170 (39.4) | 100(23.2) |
| ﻿I would be willing to carry a pad and other menstruation products for a female friend if needed | 22 (5.1) | 53 (12.3) | 19 (4.4) | 238 (55.2) | 99 (23.0) |
| ﻿Menstruation should be taught in sex education classes | 4 (0.9) | 4 (0.9) | 4 (0.9) | 230 (53.4) | 189(43.9) |
| ﻿Men can understand menstrual pains[cramps] and mood swings | 25 (5.8) | 78 (18.1) | 31 (7.2) | 196 (45.5) | 101(23.4) |
