# Supplementary material for: Adolescent boys’ sociocultural beliefs and attitudes toward menstruation in selected high schools in Ghana: Mediation and moderation effect of knowledge
Source: PLOS Glob Public Health. 2025 Jun 5;5(6):e0004354. doi: 10.1371/journal.pgph.0004354 (PMC12140258; doi:10.1371/journal.pgph.0004354)
Supplement: Attachment — Submitted filename: Response letter.docx [file pgph.0004354.s006.docx]

**Department of Epidemiology and Biostatistics**

Telegrams and cables:

Telephone: +233 (0) 362 196122

My ref:

Your ref: PMB 31, Ho - Volta Region

April 24, 2025.

Editors-in-Chief

PLOS Global Public Health

1875 Mission Street

Suite 103 #188 San Francisco, CA 94103

United States

Dear Editors,

Thank you for the opportunity to revise our paper. We have carefully considered the comments and provided responses below.

| **Reviewer** | **Reviewer comment** | **Authors’ response** |
| --- | --- | --- |
| 1 | **Comment #1:**   You tried to show that knowledge has significant effects on sociocultural beliefs. How about the reverse? not explained (considered). There are conditions that socio-cultural beliefs can interfere scientific facts, knowledge and attitude. So, it is better to revise the conceptual framework of the study. | **Revision on pp.15**  Thank you for your comment regarding the potential for sociocultural beliefs to influence knowledge and attitudes. We fully acknowledge that the relationship between knowledge and sociocultural beliefs can be bidirectional. Indeed, there is ample evidence suggesting that deeply rooted sociocultural norms and beliefs can shape the way individuals perceive, interpret, and even accept scientific knowledge, particularly in sensitive areas such as menstruation.  However, in this study, we intentionally conceptualized knowledge as a potential mediator and moderator in the relationship between sociocultural beliefs and attitudes. This decision was grounded in the theoretical framework guiding our work, which draws on behavior change models that position knowledge acquisition as a crucial step toward transforming attitudes and behaviors. Additionally, our study seeks to contribute to intervention design, where improving knowledge is often one of the more immediately actionable components in educational and policy efforts.  While we agree that future research should explore the reverse pathway, we chose to maintain our current conceptual direction to align with the study’s objectives and to offer targeted recommendations for educational and health promotion strategies.  We have now clarified this rationale in the manuscript under the strength and limitation section (pp.15) and have noted the bidirectional nature of the relationship as an important consideration for future studies. |
| 1 | **Comment #2:**   On the methodology part, it is better to describe the specific type of statistical test you used rather than describing by the type of model used. The sample size determination considered a prevalence of 50%, however there are previous reports of similar studies. The sample size using different variables also should have been extrapolated. | **Revisions on pp.5**  The authors introduced the use of **multivariate regression analysis** just before the SEM to provide additional context. We, however, believe that describing the model used enhances transparency and supports other researchers in understanding and potentially replicating our approach.  Regarding the sample size determination, we employed a definitive population formula with an assumed prevalence of 50%. This assumption was maintained to ensure a conservative estimate, maximizing the required sample size and thus enhancing the statistical power and representativeness of our study. |
| 1 | **Comment #3:** Inclusion and exclusion criteria are not mentioned. | **Revisions on pp. 4**  The authors have indicated the eligibility criteria. |
| 1 | **Comment #4:** The ethical issues, consents and privacy protocols for study participants are not clearly indicated. (mandatory) | **Revision on pp. 6**  The authors have introduced a section “Ethical consideration” which covers all ethical issues. |
|  | | |
| 2 | **Comment #1:** The manuscript is technically sound and the data supported the conclusion that was drawn. The manuscript data however, weren't provided right away but the corresponding author has indicated that data will be made available on request. The authors have also provided supporting information at the end of the manuscript's pdf file. The manuscript therefore, meets the PLOS Global Public Health publication criteria. | **No revision required** |

We appreciate your time and consideration and look forward to the possibility of contributing to PLOS Global Public Health. Please address all correspondence regarding this manuscript to Israel Wuresah at [iwuresah1996@gmail.com](mailto:iwuresah1996@gmail.com); [iwuresah22pg@sph.uhas.edu.gh](mailto:iwuresah22pg@sph.uhas.edu.gh).

Sincerely,

Israel Wuresah
